# Non alcoholic fatty liver disease increases the mortality from acute coronary syndrome: an observational study from Sri Lanka

**DOI:** 10.1186/s12872-016-0212-8

**Published:** 2016-02-12

**Authors:** Nilanka Perera, Jegarajah Indrakumar, Waruni Vijitha Abeysinghe, Vihangi Fernando, W. M. C. K. Samaraweera, Jayamal Sanjaya Lawrence

**Affiliations:** Department of Medicine, University of Sri Jayewardenepura, Nugegoda, Sri Lanka; Department of Radiology, Colombo South Teaching Hospital, Kalubowila, Sri Lanka

**Keywords:** Non alcoholic fatty liver disease, Acute coronary syndrome, Mortality, Sri Lanka

## Abstract

**Background:**

Non alcoholic fatty liver disease is an independent risk factor for coronary artery disease. But its effect on acute coronary syndrome is not clear. We performed this study to identify the prevalence of NAFLD in patients with ACS admitted to a tertiary care center in Sri Lanka. We also described the association of NAFLD with the severity of ACS predicted by the GRACE score.

**Methods:**

We performed a descriptive study including all consecutive patients with non-fatal ACS admitted to Colombo South Teaching Hospital from 01/02/2014 to 30/04/2014. Patients with excessive alcohol consumption, established cirrhosis and patients with identified risk factors for liver disease were excluded from the study. All patients underwent ultrasound scan of liver.

**Results:**

There were 120 participants, 75 (62.5 %) males and 45 (37.5 %) females with acute coronary syndrome. Average age was 61.28 ± 11.83 years. NAFLD was seen in 56 (46.7 %) patients with ACS. Patients with NAFLD had a higher GRACE score than patients without NAFLD (120.2 ± 26.9 Vs 92.3 ± 24.2, *p* < 0.001). Increased age and presence of NAFLD conferred a higher mortality risk from ACS as predicted by GRACE score. Patients with NAFLD had a higher predicted mortality during in-ward stay (adjusted OR 31.3, CI 2.2–439.8, p = 0.011) and at 6 months after discharge (adjusted OR 15.59, CI 1.6–130.6, p = 0.011).

**Conclusions:**

Patients with NAFLD have a higher predicted mortality from acute coronary syndrome and thus require aggressive treatment of CAD. It is important to consider this novel risk factor when risk stratifying patients with ACS.

## Background

Non alcoholic fatty liver disease (NAFLD) is the hepatic manifestation of metabolic syndrome [1, 2]. It could progress to more aggressive non alcoholic steatohepatitis, cirrhosis and hepatocellular carcinoma [3]. Macrovesicular steatosis is the characteristic histological finding. The prevalence of NAFLD has doubled over the last 20 years becoming the number one cause of liver disease in the western countries [4]. It is an emerging problem in the South Asian countries despite having lower body mass index. Sri Lanka is facing this growing epidemic of NAFLD and a recent population based study reported a prevalence of 32.6 % in an urban predominantly sedentary population [5].

Insulin resistance, dyslipidaemia and obesity coexist with NAFLD and these factors predispose to atherosclerosis. Longitudinal studies have shown that cardiovascular disease is one of the important causes of morbidity and mortality in patients with NAFLD. However, emerging evidence suggest that the risk of coronary artery disease in NAFLD is independent of other metabolic risk factors. These patients have increased carotid artery intimal thickness, lower endothelial flow mediated vasodilatation, increased coronary calcium score and surrogate markers of early atherosclerosis [6, 7]. The exact mechanism is still unclear, but studies postulate that increase activity of inflammatory mediators [8–10], reduced endothelial function, reduced adiponectin levels [11], reduced collateral circulation [12], reduced vascular repair capacity [13] and atherogenic lipoprotein profile contribute to coronary artery disease in these patients.

There is evidence that presence of NAFLD causes more severe coronary artery disease. Studies have evaluated the coronary plaque burden by angiography or multi slice CT. Patients with NAFLD are known to have more complex coronary artery disease in angiography [14]. But the effect on clinical outcome and mortality is not known. Furthermore, the impact of NAFLD on acute coronary syndrome (ACS) is not clear. Presence of such an association would necessitate the need to screen and manage coronary artery disease aggressively in patients with NAFLD. Therefore we performed this study to compare the predicted mortality and the clinical severity of acute coronary syndrome in patients both with and without NAFLD.

We investigated the prevalence of NAFLD in patients with ACS admitted to a tertiary care center in Sri Lanka, the association of NAFLD with ACS and its effect on the severity of ACS as predicted by the GRACE score.

## Methods

This was a descriptive study performed in the University medical unit at Colombo South Teaching hospital from 01^st^ February 2014 to 30^th^ April 2014.

### Study population

All consecutive patients with non fatal acute coronary syndrome admitted during the study period were included. Inclusion criteria were; a) history of chest pain at rest or other symptoms suggestive of an ACS with the most recent episode occurring within 24 h of admission and b) compatible ECG changes and/or positive cardiac biomarkers.

Patients with the following features were excluded from the study; a) Participants with an increased alcohol intake defined as >20 g/day during the last 2 years elicited in the history by the interviewer, b) Patients with cirrhosis diagnosed prior to admission or after admission based on the liver profile and ultrasound scan of liver, c) Patients with known risk factors for liver disease, d) Patients who died following admission before evaluation of NAFLD, e) use of agents known to induce fatty liver such as steroids, chemotherapeutic agents, amiodarone within the previous 6 months.

All patients were interviewed to obtain demographic data and quantify the alcohol consumption.

### Baseline variables

Anthropometric measures such as weight, height, body mass index (BMI) and waist circumference were measured. Obesity was defined as BMI ≥ 27.5 kg/m^2^. High waist circumference was taken as >90 cm in males and >80 cm in females. Fasting blood was tested for blood glucose, total cholesterol, serum triglyceride and LDL cholesterol. ALT level was measured. Diabetes was defined as two FBS values >5.6 mmol/L, HbA1c > 6.5 % or an already diagnosed case; hypertension was defined as blood pressure > 140/90 mmHg or a previously diagnosed case.

### Diagnosis of NAFLD

Although liver biopsy is the gold standard test to diagnose NAFLD, it was not performed due to the invasive nature of the test and ethical concerns. Ultrasonography has a sensitivity of 90 % and a specificity of 95 % when hepatic fat infiltration detected by liver biopsy is greater than 33 % [15]. It is a non invasive test widely adopted for screening. Ultrasound scan of the liver was performed by two experienced radiologists. Fatty liver was diagnosed based on widely accepted criteria; increased echogenicity of liver in comparison with the right renal cortex, attenuation of the ultrasound beam and poor visualization of intra hepatic structures [16]. Patient was diagnosed to have NAFLD if both operators agreed on the diagnosis. All the ultrasound scans were performed by two experienced radiologists to minimize inter-operator variability.

### Severity of acute coronary syndrome: GRACE score

GRACE score was calculated in all patients on admission to assess severity of ACS. It takes in to account eight independent risk factors for in-hospital death and post-discharge death up to 6 months and is a useful predictor of outcome in ACS [17, 18]. Risk of short term mortality ranges from 2 % in low risk categories to >11 % in high risk groups. This categorization was used in our study to predict mortality during hospital stay and up to 6 months after discharge [17, 18].

### Statistical analysis

Statistical analysis was performed using SPSS 17.0 version. Continuous variables were described as mean ± standard deviation. Number of cases and percentages were given for categorical variables. Chi square test was used to find the association between categorical variables and student t-test for univariate analysis of normally distributed continuous variables and Mann Whitney U test for variables with a skewed distribution. Intermediate and high risk categories according to GRACE score were grouped together and logistic regression was adopted to find the covariates associated with a worse outcome in ACS. Multiple logistic regression was used to eliminate the effect of confounding variables. For multivariate analysis, parameters having associations with collateral development with *p*-values <0.10 were entered into the analysis. Statistical significance was defined as *p* < 0.05.

### Ethical approval

Ethical approval was obtained by the ethics review committee of University of Sri Jayewardenepura. Informed consent was obtained from all study participants and those who did not give consent were excluded.

## Results

There were 120 participants with ACS, 75 (62.5 %) males and 45 (37.5 %) females with an average age of 61.28 ± 11.83 years. NAFLD was identified in 56 (46.7 %). Demographic data and metabolic risk factor profile is depicted in Table 1.

**Table 1:** Demographic variables and metabolic risk factor profile in patients with NAFLD and without NAFLD

Variable	NAFLD	Non- NAFLD	Total	Significance
	n = 56	n = 64	n = 120	
Age(years)	62.9 ± 10.8	59.8 ± 12.6	61.28 ± 11.8	*p = 0.157*
Gender				
Male	30 (53.6)	45 (70.3)	75 (62.5)	*p = 0.059*
Female	26 (46.4)	19 (29.7)	45 (37.5)	
Diabetes	38 (67.9)	32 (50.0)	70 (58.3)	***p = 0.048***
Hypertension	44 (78.6)	40 (62.5)	84 (70.0)	*p = 0.055*
Dyslipidaemia	29 (51.8)	21 (32.8)	50 (41.7)	***p = 0.035***
Smoking	24 (42.9)	31 (48.4)	55 (45.8)	*p = 0.815*
Alcohol consumption				
Never	33 (58.9)	37 (57.8)	70 (58.3)	*p = 0.902*
< 20 g/day	23 (41.1)	27 (42.2)	50 (41.7)	
BMI	24.59 ± 3.5	24.69 ± 12.9	24.64 ± 9.8	*p = 0.958*
Obesity (BMI > 27.5 kg/m2)	12 (21.5)	03 (4.7)	15 (12.5)	***p = 0.005***
Waist circumference	101.2 ± 13.6	101.4 ± 17.3	101.3 ± 15.6	*p = 0.941*
Increase waist circumference ^a^	48 (85.7)	49 (76.6)	97 (80.8)	*p = 0.204*
FBS	156.7 ± 72.9	134.7 ± 72.0	144.6 ± 72.9	*p = 0.111*
Total Cholesterol	213.6 ± 40.3	189.3 ± 26.4	200.6 ± 35.6	***p < 0.001***
Triglyceride	159.1 ± 46.4	122.2 ± 46.8	139.9 ± 49.9	***p < 0.001***
LDL-cholesterol	148.2 ± 36.2	129.5 ± 24.2	138.6 ± 31.9	***p = 0.005***
ALT	62.9 ± 46.2	29.4 ± 11.9	44.9 ± 36.5	***p < 0.001***
Type of ACS				
STEMI	16 (28.6)	16 (25.0)	32 (26.7)	*p = 0.659*
Non-STEMI	40 (71.4)	48 (75.0)	88 (73.3)	

^a^ Waist circumference >90 cm in males and >80 cm in females were considered high. Significant associations are given in bold. BMI, body mass index; FBS, fasting blood sugar; LDL, low density lipoprotein; ALT, Alanine transaminase

Majority of patients (n = 117, 97.5 %) had an uneventful recovery while only 02 (1.7 %) patients had persistent angina and 01 (0.8 %) underwent urgent revascularization. Based on GRACE score all patients were categorized as low-risk, intermediate risk and high-risk according to standard criteria. GRACE score in patients with NAFLD and without NAFLD is given in Table 2.

**Table 2:** GRACE score and the risk categories in patients with NAFLD and without NAFLD

ACS severity	NAFLD	Non-NAFLD	Total	Significance
	*n* = 56	*n* = 64	*n* = 120	
GRACE score Mean ± SD	120.2 ± 26.9	92.3 ± 24.2	105.6 ± 29.0	*p < 0.001*
Predicted In-hospital mortality				
Low risk	22 (39.3)	48 (75.0)	70 (58.3)	*p < 0.001*
Intermediate risk	19 (33.9)	9 (14.1)	28 (23.3)	
High risk	12 (21.4)	2 (3.1)	14 (11.7)	
Predicted post discharge mortality at 6 months				
Low risk	7 (12.5)	28 (43.8)	35 (29.2)	*p < 0.001*
Intermediate risk	21 (37.5)	23 (35.9)	44 (36.7)	
High risk	25 (44.6)	8 (12.5)	33 (27.5)	

Patients in high and intermediate risk of death during the hospital stay and at 6 months were grouped together. This group was compared with the low risk group. Logistic regression was used to identify the factors associated with an intermediate-high risk (Table 3 and Table 4). Presence of diabetes was associated with a higher risk of in hospital death from ACS and 6 months after discharge. This effect was not seen after correcting for other confounding factors. Female gender was associated with a worse outcome but did not have a significant effect after correcting for other covariates.

**Table 3:** Risk factors associated with a predicted intermediate-high risk of death from ACS during hospital stay according to GRACE score (Multivariate logistic regression)

Variable	OR (CI)	Significance	Adjusted OR (CI)	Significance
NAFLD	6.15 (2.6–14.4)	*p < 0.001*	31.3 (2.2–439.8)	***p = 0.011***
Age	1.15 (1.1–1.2)	*p < 0.001*	1.32 (1.1–1.5)	***p = 0.001***
Female gender	8.77 (3.6–21.1)	*p < 0.001*	2.94 (0.4–20.4)	*NS*
Diabetes	2.36 (1.04–5.3)	*p = 0.039*	5.85 (0.6–53.6)	*NS*
Hypertension	3.14 (1.2–8.1)	*p = 0.017*	1.35 (0.17–10.9)	*NS*
Dyslipidaemia	3.43 (1.5–7.6)	*p = 0.003*	2.16 (0.3–14.5)	*NS*
Smoking	0.29 (0.13–0.67)	*p = 0.003*		*NS*
Obesity	5.0 (1.4–17.2)	*p = 0.011*	1.03 (0.1–10.5)	*NS*
LDL	1.02 (1.003–1.034)	*p = 0.017*	1.01 (0.97–1.05)	*NS*
ALT	1.03 (1.01–1.05)	*p = 0.003*	1.04 (0.98–1.09)	*NS*
Waist circumference	1.01 (0.9–1.04)	*p = 0.374*		

LDL, low density lipoprotein; ALT, Alanine transaminase; NS, not significant. Significant associations are given in bold

**Table 4:** Risk factors associated with a predicted intermediate-high risk of death from ACS 6 months post discharge according to GRACE score (Multivariate logistic regression)

Variable	OR (CI)	Significance	Adjusted OR (CI)	Significance
NAFLD	5.93 (2.3–15.3)	*p < 0.001*	15.59 (1.6–130.6)	***p = 0.011***
Age	1.26 (1.2–1.4)	*p < 0.001*	1.34 (1.1–1.5)	***p < 0.001***
Female gender	4.03 (1.5–10.8)	*p = 0.006*	0.45 (0.07–3.1)	*NS*
Diabetes	1.32 (0.58–2.9)	*p = 0.501*		
Hypertension	1.58 (0.7–3.7)	*p = 0.294*		
Dyslipidaemia	2.19 (0.9–5.2)	*p = 0.073*	2.08 (0.42–10.3)	*NS*
Obesity	7.49 (0.9–59.8)	*p = 0.057*	0.56 (0.04–7.1)	*NS*
LDL	1.00 (0.9–1.02)	*p = 0.369*		
SGPT	1.02 (1.00–1.05)	*p = 0.026*	1.01 (0.9–1.09)	*NS*
Waist circumference	0.99 (0.96–1.02)	*p = 0.480*		

*LDL* low density lipoprotein, *ALT* Alanine transaminase, *NS* not significant. Significant associations are given in bold

## Discussion

This study evaluated the effect of NAFLD on predicted mortality from ACS. Previous studies have compared angiographic or multi slice CT data as a measure of coronary artery disease (CAD) burden in patients with NAFLD. Functional significance of NAFLD-related coronary lesions is not proved and studies in patients with acute coronary syndromes (ACS) are lacking [19, 20]. Furthermore, mortality data and data on South Asian populations are sparse.

We found a NAFLD prevalence of 46.7 % among our study participants with ACS. It is higher than the previously reported community prevalence of NAFLD in Western countries [21] and in Sri Lanka [5]. This is an expected finding with the accumulating evidence of a strong association of NAFLD and CAD. In addition, our study population represented a group with increased metabolic risk factors compared to healthy adults. NAFLD prevalence in our study is comparable to data from China reporting a CT detected NAFLD prevalence of 45.8 % [6]. However, two studies conducted in western countries incorporating patients with ACS revealed a NAFLD prevalence exceeding 80 % using Ultrasonography [14, 20]. One reason could be due to the increase prevalence of obesity in these countries than Sri Lanka which is a known predisposing factor for NAFLD. Our population had an average BMI of 24.6 and only 12.5 % were obese. More than 80 % had a higher than normal waist circumference reflecting the higher prevalence of central obesity in Sri Lanka. However, waist circumference was not associated with NAFLD or with high mortality from ACS.

Diabetes, dyslipidaemia, hypertension were commoner in the NAFLD group which is comparable to published literature [5]. There was a non significant increase in NAFLD among females in our study.

Our data reveal a significantly higher GRACE score in patients with NAFLD compared to patients without NAFLD (Table 2). More patients with NAFLD were seen in the high and intermediate risk groups. Further analysis revealed that presence of fatty liver is independently associated with a worse outcome. Mortality predicted by the GRACE score during the hospital stay and post discharge up to 6 months was high among NAFLD patients with an adjusted OR of 31 and 15.6. This association persisted after eliminating the effect of metabolic risk factors and age. This study shows that age and NAFLD are an important factors contributing to cardiovascular disease related deaths. A study conducted previously reported that cardiovascular death, non fatal myocardial infarction and need for revascularization was not different in patients with NAFLD versus patients without NAFLD during a follow up of 87 ± 22 weeks [22]. Long term follow up data of our cohort would also provide useful information on the number of actual adverse cardiac events in this group.

Important findings of note include the effect of female gender on intermediate-high risk of mortality from ACS. Previous studies have demonstrated that males are at risk of severe CAD [6]. However the effect of gender on CAD is controversial since a descriptive study found that coronary collateral circulation was poor in females which lost its significance when corrected for confounders [12]. Our results indicate that female gender was associated with increased mortality, and this effect was most likely due to the higher prevalence of risk factors in females. Presence of diabetes, hypertension, smoking, dyslipidaemia and obesity contributed to mortality but the effect was not seen on multivariate analysis.

### Study limitations

Main limitation was that we used ultrasonography to diagnose NAFLD . Although liver histology is the gold standard, the invasiveness of the procedure limits its use. Using liver histology as a standard, the positive predictive value of ultrasonography ranges from 77 to 96 %, and the negative predictive value ranges from 65 to 87 % [23, 24, 25]. Although our study sample was small, we were able to give important inferences in our study.

## Conclusions

Patients with NAFLD have a higher predicted mortality from acute coronary syndrome and thus require aggressive treatment of CAD. We strongly believe that it is important to consider this novel risk factor when risk stratifying patients with ACS.

## References

[1] Marchesini G, Brizi M, Bianchi G, Tomassetti S, Bugianesi E, Lenzi M (2001). Nonalcoholic fatty liver disease: a feature of the metabolic syndrome. Diabetes.

[2] Marchesini G, Bugianesi E, Forlani G, Cerrelli F, Lenzi M, Manini R (2003). Nonalcoholic fatty liver, steatohepatitis and the metabolic syndrome. Hepatology.

[3] Day CP (2002). Pathogenesis of steatohepatitis. Best Pract Res Clin Gastroenterol.

[4] World Gastroenterology Organization (2012). WGO Global Guidelines NAFLD/NASH (long version) Nonalcoholic Fatty Liver Disease and Nonalcoholic Steatohepatitis.

[5] Dassanayake AS, Kasturiratne A, Rajindrajith S, Kalubowila U, Chakrawarthi S, De Silva AP (2009). Prevalence and risk factors for non-alcoholic fatty liver disease among adults in an urban Sri Lankan population. J Gastroenterol Hepatol.

[6] Ling S, Shu-zheng LU (2011). Association between non-alcoholic fatty liver disease and coronary artery disease severity. Chin Med J.

[7] Kim D, Choi S, Park EH, Lee W, Kang JH, Kim W (2012). Nonalcoholic Fatty Liver Disease Is Associated With Coronary Artery Calcification. Hepatology.

[8] Schindhelm RK, Dekker JM, Nijpels G, Bouter LM, Stehouwer CD, Heine RJ (2007). Alanine aminotransferase predicts coronary heart disease events: a 10-year follow-up of the Hoorn Study. Atherosclerosis.

[9] Targher G, Bertolini L, Poli F, Rodella S, Scala L, Tessari R (2005). Nonalcoholic fatty liver disease and risk of future cardiovascular events among type 2 diabetic patients. Diabetes.

[10] Tarantino G, Costantini S, Finelli C, Capone F, Guerriero E, La Sala N (2014). Carotid Intima-Media Thickness Is Predicted by Combined Eotaxin Levels and Severity of Hepatic Steatosis at Ultrasonography in Obese Patients with Nonalcoholic Fatty Liver Disease. PLoS One.

[11] Choi DH, Lee SJ, Kang CD, Park MO, Choi DW, Kim TS (2013). Nonalcoholic fatty liver disease is associated with coronary artery disease in Koreans. World J Gastroenterol.

[12] Arslan U, Kocao˘glu I, Balcı M, Duyuler S, Korkmaz A (2012). The association between impaired collateral circulation and non-alcoholic fatty liver in patients with severe coronary artery disease. J Cardiol.

[13] Chiang CH, Huang PH, Chung FP, Chen ZY, Leu HB, Huang CC (2012). Decreased Circulating Endothelial Progenitor Cell Levels and Function in Patients with Nonalcoholic Fatty Liver Disease. PLoS One.

[14] Agaç MT, Korkmaz L, Cavusoglu G, Karadeniz AG, Agaç S, Bektas H (2013). Association between non alcoholic fatty liver disease and coronary artery disease complexity in patients with acute coronary syndrome: a pilot study. Angiology.

[15] Hamaguchi M, Kojima T, Takeda N, Nagata C, Takeda J, Sarui H (2007). Nonalcoholic fatty liver disease is a novel predictor of cardiovascular disease. World J Gastroenterol.

[16] Targher G, Bertolini L, Rodella S, Lippi G, Franchini M, Zoppini G (2008). NASH predicts plasma inflammatory biomarkers independently of visceral fat in men. Obesity (Silver Spring).

[17] Granger CB, Goldberg RJ, Dabbous O, Pieper KS, Eagle KA, Cannon CP (2003). Predictors of hospital mortality in the global registry of acute coronary events. Arch Intern Med.

[18] Eagle KA, Lim MJ, Dabbous OH (2004). A validated prediction model for all forms of acute coronary syndrome: estimating the risk of 6- month postdischarge death in an international registry. JAMA.

[19] Bathia LS, Curzen NP, Calder PC, Byrne CD (2012). Non-alcoholic fatty liver disease: a new and important cardiovascular risk factor?. Eur Heart J.

[20] Boddi M, Tarquini R, Chiostri M, Marra F, Valente S, Giglioli C (2013). Nonalcoholic fatty liver in non diabetic patients with acute coronary syndromes. Eur J Clin Invest.

[21] Loria P, Adinolfi LE, Bellentani S, Bugianesi E, Grieco A, Fargion S (2010). Practice guidelines for the diagnosis and management of nonalcoholic fatty liver disease. A decalogue from the Italian Association for the Study of the Liver (AISF) Expert Committee. Dig Liver Dis.

[22] Wong VW, Wong GL, Yip GW, Lo AO, Limquiaco J, Chu WC (2011). Coronary artery disease and cardiovascular outcomes in patients with non-alcoholic fatty liver disease. Gut.

[23] Graif M, Yanuka M, Baraz M, Blank A, Moshkovitz M, Kessler A (2000). Quantitative estimation of attenuation in ultrasound video images: correlation with histology in diffuse liver disease. Invest Radiol.

[24] Mathiesen UL, Franzen LE, Aselius H, Resjo M, Jacobsson L, Foberg U (2002). Increased liver echogenicity at ultrasound examination reflects degree of steatosis but not of fibrosis in asymptomatic patients with mild/moderate abnormalities of liver transaminases. Dig Liver Dis.

[25] Palmentieri B, de Sio I, La Mura V, Masarone M, Vecchione R, Bruno S (2006). The role of bright liver echo pattern on ultrasound Bmode examination in the diagnosis of liver steatosis. Dig Liver Dis.

